# The enzyme toilet rim block ‘pCure’ does not efficiently remove drug residues in a hospital setting - exemplifying the importance of on-site implementation testing

**DOI:** 10.1080/20008686.2018.1553463

**Published:** 2018-12-06

**Authors:** Sofia Svebrant, Therese Olsen, Jim Larsson, Patrik Öhagen, Hanna Söderström, Josef D. Järhult

**Affiliations:** a Uppsala University Hospital, Uppsala County Council, Uppsala, Sweden; b Uppsala Clinical Research Center, Uppsala University, Uppsala, Sweden; c Department of Public Health and Clinical Medicine, Occupational and Environmental Medicine, Umeå University, Umeå, Sweden; d Zoonosis Science Center, Department of Medical Sciences, Uppsala University, Uppsala, Sweden

**Keywords:** API, enviroment, antibiotic resistance, waste water, pharmaceuticals, sewage treatment

## Abstract

**Introduction:** Negative environmental effects of active pharmaceutical ingredients (APIs) are increasingly recognized, especially concerning antibiotics, and hospitals are important point sources. “pCure” is a toilet rim block containing API-degrading enzymes; the producing company claims positive in vitro results but no implementation studies have been performed. **Materials and methods:** In a university hospital setting, 16 weeks were randomized to installation or no installation of pCure in all 261 toilets connected to the same cesspit where sewage water was sampled daily. Ninety-six samples were analyzed for 102 APIs using liquid chromatography/tandem mass spectrometry. **Results and Discussion:** Fifty-one APIs were detected with a large variation in levels but no significant differences in the initial statistical analysis. More statistical testing of API level ratios (pCure installed/not installed) yielded some cases of significant decrease. Differences were small and not consistent when comparing means and medians. We cannot exclude a small pCure effect but clearly pCure has no effect of biological importance. **Conclusion:** pCure is not useful to reduce drug residue discharge in a hospital setting. In a bigger perspective, our study exemplifies that products claiming to reduce an environmental problem need to be tested in on-site implementation studies by independent researchers before reaching the market.

## Introduction

Waste water treatment plants (WWTPs) are generally not designed to remove pharmaceutical residues (or active pharmaceutical ingredients, APIs) and most of these substances pass the plant unaffected [1], and reach the aquatic environment through discharge from WWTPs [2]. APIs excreted from patients using pharmaceuticals constitute the largest input of APIs to WWTPs [3]. In the recipient, APIs constitute a risk to the aquatic and terrestrial ecosystems and, in the long run, also a health risk to humans [4].

Spread of antibiotic resistance through the environment is a major problem [5]. Today, safe levels of antibiotics and other APIs in the environment are not known. Bengtsson-Palme & Larsson [5], have shown that even very low antibiotic levels drive resistance development. It is also shown by Gullberg et al. [6], that this is happening in vitro, at antibiotic concentrations several hundred times below the MIC (minimal inhibitory concentration), similar to concentrations in waste water from hospitals [7]. Thus, it is important to ensure that the API levels in the environment stay as low as possible [7].

Municipal waste water is the main contributor of APIs to WWTPs, but hospitals constitute an important point source [1]. High levels of antibiotics (including last-line drugs) are found in the hospital sewage water together with antibiotic resistant bacteria, as well as wild-type bacteria that can serve as recipients for horizontal transfer of resistance. Today, no specific treatment of hospital sewage water is performed in Sweden or most other countries, and the sewage is discharged to municipal sewage treatment as is. For comparison, in a study of a Norwegian hospital (1200 beds), roughly the same size as our study site Uppsala university hospital (UUH) (1000 beds), mean pharmaceutical concentrations in effluent waste water from the hospital measured in 24-hour samples were: Ciprofloxacin 23,336 ng/l, Doxycycline 124 ng/l, Diclofenac 819 ng/l, Metoprolol 1072 ng/l and Trimetoprim 4302 ng/l [3].

Thus, reducing the levels of antibiotics and/or antibiotic resistant bacteria already in hospitals will lower the risk of antibiotic resistance in the sewer system, WWTPs, and the recipient [7]. Also inside hospitals, resistance can be problematic as effluent waste water contains resistant bacteria, and resistance (genes) can be transferred from harmless commensals to pathogenic bacteria and then spread within the hospital through toilets, sinks, cesspits, and/or air [8].

According to Casas et al. [9], some hospitals around the world have installed different types of sewage treatment to reduce levels of APIs. For example, in hospitals in China and Japan, there is treatment on-site via membrane bio reactors (MBRs) or conventional waste water treatment [10]. Other examples are hospitals in Luxembourg, Germany, Netherlands, Switzerland [11] and Denmark [12] that have an on-site waste water treatment facility. It is common to use MBR as a first step and then use an extra step, e.g. activated carbon, osmosis, nanofiltration or some oxidation process to remove the rest of the APIs [9]. A study at Aarhus university hospital (2015) tested, for the first time, to treat the hospital waste water with MBBR (Moving bed biofilm reactor) technology, as the only treatment after a mechanically pre-treated wastewater. The result showed that the removal efficiency differed depending of type of API, from around 10 % to 100 % removal [9]. At Herlevs hospital in Denmark, a full scale treatment for waste water was installed in 2014. The treatment includes an MBR-reactor and then steps of ozone treatment and GAC-filter (granulated active carbon) followed by UV-irradiation. After the treatment, the water is clean enough to be released directly to the recipient (99,9% API removal) [13]. However, all these approaches are very resource demanding due to cost and technical complexity.

A novel approach to the problem is presented by the product ‘pCure’ (Pharem Biotech, Stockholm, Sweden) which is a toilet rim block that contains enzymes designed to degrade APIs. PCure could become an important tool to reduce the discharge of APIs in many settings due to the simplicity, flexibility and low cost (approximately USD 5–10 per block). The producing company claims positive in vitro results and for a brief period, pCure was marketed to consumers in Swedish pharmacies. However, to our knowledge there are no published studies demonstrating effect of enzymes to degrade APIs, and no published implementation studies demonstrating effect of pCure. Given the potential of pCure and the importance of on-site implementation to demonstrate effect, we decided to perform an implementation study of pCure in a hospital setting.

## Materials and methods

### Pcure

For this study, we bought 2088 ‘pCure Hospital’ blocks from Pharem Biotech (Stockholm, Sweden, batch no 029915). Pharem Biotech was not involved in the planning, execution, or evaluation of the study. PCure is a toilet block placed over the rim of the toilet, consisting of 40 % starch, 40 % cellulose, 5 % magnesium stearate, 10 % Tween-20 and 5 % enzymes. The mixture is pressed to form a block and then put in a plastic casing. Every time the toilet is flushed, an expected amount of 2 % of the total block volume is released into the water by flushing over the block. The exact enzyme content in the blocks and the structure of the enzymes were unknown to the authors of this study. The enzymes are expected to stay latent in the still toilet water between flushes and follow the water down the drain, together with the newly released enzymes from the last flushing. Since enzymes are proteins, they will quickly be degraded by the active biological environment in the sewage.

### Experimental set-up

Uppsala University Hospital (UUH) is a full-scale tertiary/university hospital with 1000 beds. In the hospital area, there are three cesspits and for this study one of them was chosen. It has a flow of 2–4m^3^/h and 261 toilets in five buildings are connected to it. The buildings contain inpatient wards and outpatient clinics for oncology, plastic surgery and otorhinolaryngology, operating rooms and other outpatient departments, as well as facilities for administrative staff, construction workers, and a few public toilets. Inside the cesspit an automated sampler was installed that sampled 50 ml of sewage water 8 times per hour during weekdays, and 36 times per hour during weekends (different settings due to large difference in flow). The automated sampler was emptied in the morning (sometime between 7–9 am) every day of the week except for Saturday (corresponding to the water from Friday morning – Saturday morning, which was a wash-out day as pCure blocks were inserted/removed Fridays). The study comprised of 16 weeks from March to June 2018. Each week was randomized to either have pCure blocks installed in all 261 toilets the entire week, or to have no blocks installed at all. The randomization was performed in blocks of four, i.e. each block of four weeks contained two weeks with pCure installed and two weeks without pCure installed, but in random order. This was done to minimize risks of bias both due to systematic errors (e.g. a particular treatment being carried out every other week) and seasonal errors (e.g. more antibiotics being used during the cold season). All in all, 96 24h-samples were collected during the study, 48 from days with pCure installed, and 48 from days without pCure installed.

In the experiment cesspit, a SmartScan50 flow meter (Elmacron AB, Norrköping, Sweden) was installed, and during parts of the experiment, the sewage water flow was measured every fifth second.

### Analysis of pharmaceuticals

One hundred and two APIs were analyzed with an online solid phase extraction/liquid chromatography tandem mass spectrometry (online SPE/LC-MS/MS) system and a method previously described in detail by Lindberg et al. [14]; specific details on the on-line SPE/LC system and the MS/MS transition ions used are given by Khan et al. [15], and Grabic et al. [16], respectively. The method has been further developed both in general and to include oseltamivir carboxylate; details given by Blum et al. [17]. The analytical limit of quantification (LOQ) ranged from 1 to 20 ng/L [15]. The MS/MS method of metronidazole has not been described before: 172.0 → 128.2 (quantification ion), collision energy (CE) 14, tube lens (TL) 87 and 172.0 →s 82.3 (qualification ion), CE 26, TL 87. LOQ of 20 ng/L.

### Statistics

Fifty-one substances of the 102 analyzed were not detected in any sample and thus excluded from the statistical evaluation, leaving 51 substances to evaluate. Statistical analysis was performed using SAS 9.4 (SAS Institute Inc., Cary, NC, USA). A statistical certainty of more than 95% (i.e. a p-value <0.05) was considered significant. The statistical analysis was basically descriptive. The potential correlation patterns between repeated measurements (over time) were analyzed descriptively and graphically using scatterplots. Since the scatterplots of the repeated measures showed no strong correlation pattern, the observations were analyzed as independent. T-tests for independent samples and non-parametric Wilcoxon tests were used for each API. Data were analyzed both as ”observed cases” (i.e. considering observations below LOQ as missing values) and with observations below LOQ imputed as the LOQ value for the API in question. No adjustments for multiplicity issues were performed. The ratio of the level of each API with pCure installed divided by the level without pCure installed was evaluated to investigate if there were any trends among all APIs. If there was any trend, then the mean value of all the calculated ratios should be statistically different from one (i.e. ‘no difference’ in terms of ratio). The ratios were calculated using both the mean and the median as summary measure for the observed level of API. Ratios were also calculated on subgroups of APIs with observations >LOQ in >50%, >75%, and >95% of samples. To further assess substances where it would be most likely that pCure would have an effect, we also separately analyzed 21 substances (of which 13 were found in any of our samples) which Pharem Biotech state as examples of substances for which pCure is effective [18]. We used both t-tests (a parametric test) and signed rank tests (a non-parametric test) to analyze the ratios.

## Results

The full dataset of the analysis results can be found in Supplemental Material. In Table 1, an overview of all APIs that were detected (>LOQ) in at least 25% of the samples are given.

**Table 1.** Descriptive statistics of the 36 APIs detected >LOQ (limit of quantification) in at least 25% of samples. All API measurements are given in ng/L. n>LOQ = number of samples in which the API in question was detected (i.e. >LOQ). Low 95%CI = Lower limit of the 95% confidence interval. High 95%CI = Higher limit of the 95% confidence interval. <LOQ = LOQ = samples in which the API was not detected (i.e. <LOQ) were considered to be equal to LOQ in the statistical analysis. only >LOQ included = only samples were the API was detected (i.e. >LOQ) were included in the statistical analysis. P values <0.05 marked as bold.

	Without pCure installed				With pCure installed				T-test (parametric)		Wilcoxon (non-parametric)	
API	n > LOQ	Mean	Low 95%CI	High 95%CI	n > LOQ	Mean	Low 95%CI	High 95%CI	p-value <LOQ = LOQ	p-value only >LOQ included	p-value <LOQ = LOQ	p-value only >LOQ included
Alfuzosin	42	175.63	36.35	314.91	42	112.21	38.43	186.00	0.4231	0.39826	0.65759	0.73053
Amytriptyline	38	62.39	34.03	90.74	31	66.99	32.74	101.24	0.8351	0.51928	0.22738	0.88966
Atenolol	43	9648.32	4556.96	14,739.68	44	4282.34	1785.81	6778.88	0.0617	**0.04538**	0.14041	**0.03871**
Atorvastatin	38	764.77	151.12	1378.42	38	1136.03	433.69	1838.37	0.4245	0.44078	0.11568	**0.04665**
Bisoprolol	47	314.86	127.19	502.54	45	239.18	87.03	391.33	0.5310	0.56352	0.90815	0.95018
Bupropion	14	10.47	3.39	17.56	19	11.74	5.25	18.24	0.7911	0.81518	0.21515	0.59738
Carbamazepin	43	357.32	205.47	509.16	43	683.77	84.16	1283.38	0.2864	0.30014	0.94360	0.78889
Ciprofloxacin	47	23,434.92	−1614.33	48,484.18	47	29,972.29	−13,468.70	73,413.28	0.7926	0.81000	0.99109	0.85006
Citalopram	47	1091.69	596.98	1586.39	47	1112.09	286.59	1937.58	0.9659	0.99603	0.63115	0.50576
Clindamycine	47	2040.19	7.63	4072.74	44	823.30	−365.23	2011.83	0.3036	0.32942	**0.01212**	**0.02195**
Codeine	31	772.53	307.21	1237.85	31	796.53	240.68	1352.38	0.9469	0.98167	0.91220	1.00000
Desloratidin	18	65.68	36.05	95.31	18	108.44	44.20	172.68	0.2237	0.16777	0.68515	0.22319
Diclofenac	30	337.62	96.89	578.35	32	297.36	169.92	424.80	0.7682	0.60916	0.70660	0.86024
Diltiazem	16	65.63	−9.92	141.18	18	34.38	0.78	67.98	0.4522	0.34356	0.80460	0.36054
Fexofenadine	35	1864.33	340.36	3388.30	41	1738.67	237.85	3239.49	0.9062	0.67057	0.61920	0.32729
Flecainide	47	456.41	214.06	698.75	47	343.07	244.71	441.42	0.3892	0.35396	0.75173	0.61772
Fluconazole	46	4145.46	1164.98	7125.94	41	1979.44	358.58	3600.29	0.2048	0.26866	**0.02383**	0.07911
Fluoxetine	30	37.11	12.86	61.37	29	16.97	12.32	21.62	0.1075	0.10154	0.36843	0.09995
Irbesartan	19	67.77	5.41	130.13	26	267.65	−65.29	600.60	0.2334	0.37562	0.21488	0.60512
Loperamide	26	18.55	5.12	31.99	26	15.17	3.02	27.32	0.7085	0.67456	0.78146	0.37475
Metoprolol	47	2717.09	1706.33	3727.86	47	2974.95	560.17	5389.72	0.8421	0.87817	0.26578	0.19080
Mirtazapine	43	378.02	65.87	690.16	45	417.82	252.12	583.52	0.8223	0.93352	**0.04025**	0.08551
Naloxone	20	157.86	52.91	262.80	17	262.78	−104.96	630.51	0.5787	0.46662	0.58488	0.93927
Oxazepam	47	1291.92	360.46	2223.38	46	1040.01	349.39	1730.62	0.6640	0.66420	0.59715	0.58800
Paracetamol	40	35,782.51	9032.54	62,532.47	39	46,122.10	5608.03	86,636.17	0.6679	0.65944	0.66790	0.59985
Metronidazole	14	4075.24	517.16	7633.32	24	5179.35	1193.12	9165.58	0.6782	0.54678	**0.04869**	0.72784
Ranitadine	34	1335.51	444.87	2226.15	40	1291.25	418.28	2164.21	0.9432	0.64185	0.10199	0.53284
Rosuvastatin	35	1064.02	−94.03	2222.08	34	1076.75	−4.50	2158.00	0.9872	0.97853	0.50326	0.32208
Sertraline	16	30.54	16.75	44.34	19	31.57	14.64	48.49	0.9250	0.83836	0.52703	0.85549
Sulfamethoxazol	38	225,201.16	6845.58	443,556.73	42	149,501.49	−16,789.03	315,792.01	0.5814	0.47023	0.50444	0.81340
Telmisartan	20	951.38	−770.85	2673.61	24	103.13	14.20	192.05	0.3301	0.27236	0.61285	0.31647
Tetracycline	33	1627.72	−226.56	3482.01	42	3279.61	−289.32	6848.53	0.4077	0.60472	**0.00418**	0.10820
Tramadol	40	494.26	213.51	775.00	40	343.47	201.83	485.10	0.3402	0.30193	0.96133	0.90426
Trimethoprim	47	9083.34	4764.89	13,401.78	47	6836.36	2782.14	10,890.58	0.4475	0.41356	0.54903	0.43161
Venlafaxine	28	305.07	112.77	497.37	34	1068.43	11.09	2125.77	0.1521	0.23652	0.07454	0.27921
Propranolol	30	390.37	−11.43	792.16	27	139.36	58.33	220.38	0.2256	0.25825	0.46719	0.54895

Firstly, we analyzed the difference of means in the groups ‘pCure installed’ vs ‘pCure not installed’ using T-tests for independent samples and imputing the LOQ value for samples below LOQ and found no significant differences for any of the 51 APIs (Table 1). We also noted a large variation in the dataset. We then repeated the statistical testing using only observations >LOQ (i.e. treating <LOQ as missing values) and using a non-parametric test (Wilcoxon). These results are displayed in Table 1. We found seven APIs for which there were significant differences in any statistical test, but it differed if the significant difference indicated higher or lower API levels when pCure was installed. For three APIs (atenolol, clindamycin, and fluconazole), we found significantly lower levels of API when pCure was installed. For four APIs (atorvastatin, mirtazapine, metronidazole, and tetracycline) we found significantly higher API levels when pCure was installed (Table 1). There were no significant differences for any of the APIs detected in <25% of the samples using any of the statistical tests (data not shown).

Analysis of the ratios of pCure installed vs pCure not installed gave mixed results. We decided to include subgroups of APIs detected in >50%, >75%, or >90% of samples in the analysis with the idea that APIs detected in very few samples should contribute less data and thus could dilute an effect of pCure. When analyzing all APIs and subgroups we found no significant differences and ratios that ranged from 0.87 to 1.81 (a ratio of 1 indicates no effect, a lower ratio a positive effect of pCure installation and a higher ratio a negative effect) (Table 2). When repeating the analysis using only APIs for which Pharem Biotech have stated that pCure is effective [18] we found some indications of an effect of pCure (i.e. ratios statistically different from 1). Significant differences were seen using both parametric and non-parametric tests, but only when analyzing medians of levels for each API, not when analyzing means (Table 2). The ratios ranged from 0.78–2.67.

**Table 2.** Descriptive statistics of ratios ‘pCure installed/without pCure installed’. Ratios were calculated for means or medians for each API, and analysis of ratios for different subsets of APIs are summarized in the table. APIs were divided into subsets depending on the number of samples in which APIs were detected (i.e. >LOQ (limit of quantification)); >50%, >75%, and >90% of samples. The same calculations were performed using only the APIs for which Pharem Biotech state that pCure is effective. Samples <LOQ were either considered as = LOQ, or excluded from the analysis (treated as missing values). Ratio = 1 indicates no effect, ratios <1 suggest that pCure installation decreases API levels and ratios >1 suggest that pCure installation increases API levels. p Par = p-value for t-test (a parametric test). p NPar = p-value for signed rank test (a non-parametric test).

		Samples <LOQ considered as = LOQ						Only values >LOQ included in analysis					
		Means			Medians			Means			Medians		
	APIs detected in	Ratio	p Par	p NPar	Ratio	p Par	p NPar	Ratio	p Par	p NPar	Ratio	p Par	p NPar
All APIs	>90% of samples	0.90 (n = 12)	0.4325	0.3394	0.88 (n = 12)	0.2373	0.2661	0.89 (n = 12)	0.3611	0.2334	0.87 (n = 12)	0.1074	0.1514
	>75% of samples	0.97 (n = 20)	0.8013	0.4749	1.13 (n = 20)	0.3775	0.9563	0.92 (n = 20)	0.3893	0.2943	0.95 (n = 20)	0.4102	0.4524
	>50% of samples	1.02 (n = 28)	0.8535	0.3252	1.10 (n = 28)	0.3668	0.8246	0.96 (n = 28)	0.7221	0.2527	0.95 (n = 28)	0.3404	0.3142
	All samples	1.50 (n = 51)	0.2056	0.8390	1.10 (n = 51)	0.1615	0.7305	1.81 (n = 48)	0.1340	0.8007	1.23 (n = 48)	0.3920	0.3052
APIs for which Pharem Biotech states pCure is effective	>90% of samples	1.05 (n = 7)	0.7892	0.9375	0.78 (n = 7)	0.0631	0.0781	1.04 (n = 7)	0.8273	0.9375	0.83 (n = 7)	0.0325	0.0469
	>75% of samples	0.97 (n = 9)	0.8226	0.5703	0.83 (n = 9)	0.0645	0.0977	0.95 (n = 9)	0.7208	0.4258	0.85 (n = 9)	0.0174	0.0195
	>50% of samples	0.96 (n = 10)	0.7526	0.4316	0.84 (n = 10)	0.0469	0.0488	0.93 (n = 10)	0.6146	0.3223	0.85 (n = 10)	0.0117	0.0098
	All samples	2.40 (n = 13)	0.3495	0.7869	0.87 (n = 13)	0.0493	0.0488	2.67 (n = 12)	0.3581	0.5693	0.89 (n = 12)	0.0404	0.0640

When removing the pCure blocks from the toilets, we noted that in the absolute majority of cases, the blocks were still present and still contained some substance, indicating that they had not been used up.

The flow in the cesspit in the experiment varied greatly over time, with much lower flows at nighttime and during weekends. This was expected as much of the activities in the buildings included in the study only take place during daytime on weekdays. The average flow in the daytime (08.00–18.00) weekdays was 4.8 m^3^/h (n = 21 (number of days measured)), 0.84 m^3^/h daytime Saturdays, Sundays or holidays (n = 11), and 0.89 m^3^/h in nighttime (18.00–08.00, n = 18).

## Discussion

Measures to decrease discharge of drug residues from point sources are important, and the enzyme block ‘pCure’ could prove an important tool for this. However, the product has to our knowledge not been evaluated in any published studies and therefore we performed a 16-week on-site implementation study, comprising 261 toilets in a tertiary/university hospital, measuring drug residues in the sewage water daily.

In our initial statistical analysis, we did not see a significant difference for any of the APIs when pCure was installed or not. However, there was a large variation in API levels over time. We repeated the analysis using two different statistical methods (one parametric and one non-parametric) and two different assumptions regarding samples where no API was detected, i.e. <LOQ samples (to treat <LOQ samples as containing API at the LOQ level, or to treat <LOQ samples as missing values). We also analyzed ratios of mean or median levels of API with pCure installed vs not installed using one parametric and one non-parametric test. Such extensive statistical testing is not to be recommended in general, and we do acknowledge the fact that it may seem like a ‘fishing expedition’. However, we argue that in our case it is warranted as: 1) we did an initial testing using a defined statistical test and defined assumptions, and we consider the lack of significance seen in this analysis as our main finding; 2) we saw a large variation of API levels over time and thus did further testing to look for a small effect of pCure that may have been otherwise missed; and 3) we foresee the risk of other actors re-analyzing our data actively looking for indications of an effect of pCure, and we want to be as thorough and transparent as possible to prevent that.

In the further statistical evaluation, we saw seven instances where one or two of the analyses indicated a significant difference of API levels when pCure was installed as compared to when it was not. Interestingly, for only three APIs there was a significant decrease in API levels when pCure was installed, whereas for four APIs, the levels were significantly increased. There are no reasonable biological reasons why the use of pCure should increase API levels, and thus the instances of significant increase are likely due to type I error. However, this also illustrates that the similar amount of instances of significant decrease may well be due to type I errors too. Furthermore, we have not adjusted for multiplicity and such an adjustment (e.g. a Bonferroni correction) would erase all significant differences found in this study. Finally, no significant difference found in this study was significant in all tests/assumption combinations for the same difference (e.g. we found significance with a non-parametric test but not a parametric, or significance using medians but not means).

When analyzing ratios of means or medians of API levels when pCure was installed divided by levels when pCure was not installed, there were no differences when looking at all APIs or when looking at the APIs more commonly detected in the samples. When looking at only the APIs for which Pharem Biotech states that pCure is effective [18], we found significant differences when analyzing medians in some instances (using both the parametric and the non-parametric test), but never when analyzing means. The ratios were close to 1 and 0.78 was the lowest ratio detected in any analysis. We conclude that in our study, there is no evidence for an effect of pCure. Due to the large variation seen in our data and the trends seen in some of our analyses of ratios, a small effect of pCure cannot be excluded. However, our study clearly demonstrates that pCure does not have a large enough effect to be biologically meaningful in a hospital setting.

As all implementation studies, the present study has limitations. We could not control for differences in drug residues entering the experimental system, as we do not know the pharmaceutical use of patients and staff using the toilets included in the study. Differences over time in patients and/or the drugs prescribed for them and other people using the toilets likely explain the high variation seen in our dataset. However, by using block randomization of weeks with/without pCure, we believe that the risk of a systematic bias due to cyclic factors or seasonal differences has been minimized. Also, it cannot be excluded that there was some correlation between different time points and/or substances, which could have led us to overestimate the true number of data points. Further, the API analysis was performed on 24h-samples; thus parts of the sample material stayed in the sampler for up to 24 hours before being frozen. This may have allowed the enzymes in the water to elicit more degrading effect as compared to the real situation when they would be diluted and exposed to the active biological environment in the sewage downstream of the sampling point. This may have lead us to overestimate the effet of pCure.

When trying to assess and alleviate the problem of drug residues in the environment in general, and AMR induction in particular, a One Health approach is crucial [19–21]. This includes a holistic view on the human/animal/environment interface, and cooperation between several relevant disciplines such as environmental chemistry, infectious medicine, veterinary medicine, biology, ecology, microbiology, pharmacology, and behavioral sciences.

Taken together, our results strongly indicate that the product pCure is not effective in reducing waste water levels of drug residues in a hospital setting. In a bigger perspective, our study exemplifies that any product claiming to reduce an environmental problem needs to be tested in implementation studies by independent researchers before reaching the market. Continued efforts to develop and evaluate tools to lower drug residue discharge from hospitals are crucial.
